# Screening potential treatments for mpox from Traditional Chinese Medicine by using a data-driven approach

**DOI:** 10.1097/MD.0000000000035116

**Published:** 2023-09-15

**Authors:** Linyang Li, Chengchen Xu, Yinling Guo, Haozhong Wang

**Affiliations:** a College of Basic Medicine, Chengdu University of Traditional Chinese Medicine, Chengdu, China.

**Keywords:** data mining, molecular docking, molecular dynamic simulation, Mpox, network pharmacology, Traditional Chinese Medicine

## Abstract

Mpox (MPX) has escalated into a public health emergency of international concern, necessitating urgent prophylactic and therapeutic measures. The primary goal of this investigation was to systematically extract Wan Quan’s expertise in treating smallpox, as documented in Exclusive Methods for Treating Pox (Dou Zhen Xin Fa in Chinese), with the aim of identifying potential prescriptions, herbs, and components for alternative MPX therapies or drugs. This research utilized data mining to identify high-frequency Chinese Medicines (CMs), high-frequency CM-pairs, and CM compatibility rules. Network pharmacology, molecular docking, and molecular dynamic simulation were employed to reveal the potential molecular mechanisms of the core CM-pair. 119 prescriptions were extracted from Exclusive Methods for Treating Pox. We identified 25 high-frequency CMs and 23 high-frequency CM pairs among these prescriptions. Combined association rule mining analysis, Gancao (*Glycyrrhiza uralensis* Fisch.), Renshen (*Panax ginseng* C. A. Mey.), Danggui (*Angelica sinensis* (Oliv.) Diels), Shengma (*Cimicifuga foetida* L.), and Zicao (*Lithospermum erythrorhizon* Siebold & Zucc.) were selected as the core CM-pair for further investigation. Network pharmacology analysis yielded 131 active components and 348 candidate targets for the core CM-pair. Quercetin and celabenzine were chosen as ligands for molecular docking. GO and KEGG enrichment analyses revealed that the core CM-pair could interact with targets involved in immune, inflammatory, and infectious diseases. Moreover, key mpox virus targets, F8-A22-E4 DNA polymerase holoenzyme and profilin-like protein A42R, were docked well with the selected core components. And molecular dynamic simulation indicated that the component (quercetin) could stably bind to the target (profilin-like protein A42R). Our findings identified potential prescriptions, herbs, and components that can offer potential therapies or drugs for addressing the MPX epidemic.

## 1. Introduction

Mpox (MPX) is a rare viral zoonotic infectious disease, predominantly occurring in Central and West Africa, and is caused by the mpox virus (MPXV). However, From 1 January 2022 through 5 June 2023, a cumulative total of 87,929 laboratory-confirmed cases of MPX, including 146 deaths, have been reported to World Health Organization (WHO) from 111 countries/territories/areas in all 6 WHO Regions.^[1]^ This has escalated into a public health emergency of international concern, necessitating urgent prophylactic and therapeutic measures. Since there are no clinically proven specific treatments for MPXV infection,^[2]^ the use of computational chemical biology techniques to identify potential blockers of MPXV target proteins is critical in special situations, such as MPX outbreaks. For example, molecular docking, virtual screening,^[3]^ and artificial intelligence^[4,5]^ (referred to as “ dry method” research) have been widely used in TCM to prevent and treat infectious diseases. These computational methodologies are pertinent for minimizing the use of animal models in pharmacological research, assisting in the rational design of innovative and safe drug candidates, repositioning marketed drugs, and supporting medicinal chemists and pharmacologists throughout the drug discovery trajectory.^[6]^ Two primary proteins, MPXV F8-A22-E4 DNA polymerase holoenzyme^[7]^ and profilin-like protein A42R,^[8]^ have been recommended as viable targets for screening drugs that inhibit MPXV replication and proliferation, taking advantage of ongoing efforts to solve protein structures that guide structure-based drug discovery.

Both MPXV and smallpox viruses belong to the family Poxviridae and the genus Orthopoxvirus.^[9]^ The symptoms are similar to smallpox, but the disease is milder, primarily manifesting as high fever, headache, lymphadenopathy, and systemic blisters and pustules, with a case fatality rate of approximately 1% to 10%. Smallpox vaccination has been reported to offer 85% protection against MPXV, and anti-smallpox virus drugs can have anti-MPXV effects.^[10]^

**Figure 1.**
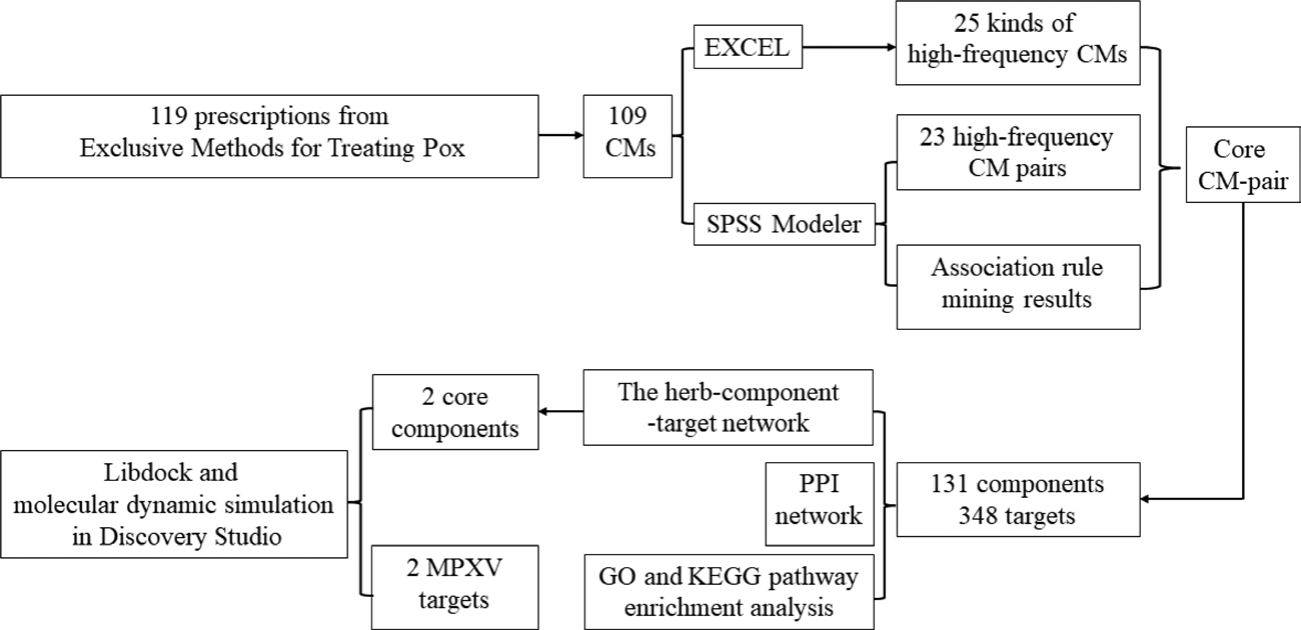
The overall framework based on an integrated screening system strategy.

Traditional Chinese Medicine (TCM), a medical system with a lengthy history, exhibits exceptional clinical efficacy against pestilence.^[11]^ TCM has a long history of foreign exchange and has now spread to 196 countries and regions, receiving increasing attention. In recent years, with the promotion of the WHO and some professional groups and people, the acceptance and recognition of Traditional medicine by the international community has gradually increased. As the representative of Traditional medicine, TCM should enhance cooperation with other Traditional medicine, and jointly promote the application and promotion of international Traditional medicine. When dealing with the epidemic situation of such infectious diseases, TCM should also work with western medicine peers to jointly promote the international application and dissemination of Chinese medicine and make contributions to human health.^[12]^ Both MPXV and smallpox viruses fall under the category of “pox” in epidemic diseases in TCM. TCM had extensive clinical experience and numerous effective prescriptions for treating smallpox, which holds referential value for MPX treatment. In June 2022, the Diagnosis and Treatment Guideline of MPX was released by the National Health Commission and the National Administration of TCM of the People’s Republics of China, recommending TCM for MPX treatment.^[13]^ Consequently, it is of great importance to explore and extract TCM experience in treating smallpox based on historical TCM classics combined with contemporary medical research methods. The present study employed data mining and an association network to identify high-frequency Chinese medicines (CMs), high-frequency CM pairs, and CM compatibility rules from ancient prescriptions.^[14]^ Subsequently, network pharmacology was applied to reveal the components, targets, and related signaling pathways of action of the core CMs-pair.^[15]^ Moreover, molecular docking and molecular dynamics simulation were utilized to investigate the molecular mechanism between core components in the core CM pair and key MPXV targets.^[16]^ The overall framework based on an integrated screening system strategy was shown in Figure 1. These results were anticipated to provide novel insights and a foundation for developing new therapies and drugs for MPX.

## 2. Materials and Methods

### 2.1. Data mining

#### 2.1.1. Data sources.

In this study, Exclusive Methods for Treating Pox (Dou Zhen Xin Fa in Chinese),^[17]^ Pharmacopoeia of the People’s Republic of China,^[18]^ Chinese Pharmacy,^[19]^ and Chinese Materia Medica^[20]^ were utilized to screen prescriptions for treating smallpox. Exclusive Methods for Treating Pox (Dou Zhen Xin Fa) was authored by Wan Quan, a renowned pediatrician from the Ming Dynasty. He extensively incorporated the experience of previous generations of doctors in treating smallpox and combined it with his own clinical experience to write this book, which recorded prescriptions for treating smallpox at various stages.^[21]^

The data preparation process comprised 2 steps: first, relevant prescription information in the book, including number, name, indication, and formula, was input into an Excel file. Second, prescription information was standardized based on the Pharmacopoeia of the People’s Republic of China and the full botanical plant names have been checked with http://www.worldfloraonline.org (last accessed on 20 Apr 2023) for subsequent data mining and association network analysis.

#### 2.1.2. Data analysis.

In this study, frequency analysis, association rule mining, and association knowledge network construction methods were employed to analyze the standardized data. Excel was used to analyze the high-frequency CMs. SPSS Modeler 18.0 software (https://www.ibm.com/cn-zh/products/spss-modeler)^[22]^ was used to construct a CMs association network, which was further visualized using Cytoscape (Version 3.9.1, available at https://cytoscape.org/)^[23]^ and analyze the compatibility rule of prescription with the apriori algorithm according to “the support ≥ 10.0%, the confidence ≥ 80.0%, and the maximum number of antecedents was 2.”

### 2.2. Potential active mechanism of the core CM-pair

Based on previous prescription information analysis results and clinical experience, 5 core CMs: Gancao (*Glycyrrhiza uralensis* Fisch.), Renshen (*Panax ginseng* C. A. Mey.), Danggui (*Angelica sinensis* (Oliv.) Diels), Shengma (*Cimicifuga foetida* L.), and Zicao (*Lithospermum erythrorhizon* Siebold & Zucc.), were selected to explore their potential mechanism. The chemical components of these CMs were obtained from TCMSP (Traditional Chinese Medicine System Pharmacology Database, http://tcmspw.com/tcmsp.php).^[24]^ Then, active components and protein targets were screened according to ADME principles of “oral bioavailability ≥ 30% and drug-likeness ≥ 0.18.”^[25]^ Furthermore, Swiss Target Prediction (http://www.swisstargetprediction.ch/)^[26]^ was used to supplement target information. Next, the UniProt website (https://www.uniprot.org/)^[27]^ was utilized to match protein targets and gene names. Subsequently, the CM-component-target network was constructed and visualized using Cytoscape (Version 3.9.1). Finally, the protein–protein interaction (PPI) network for the targets was established using the String Database (https://cn.string-db.org/)^[28]^ and was further visualized using Cytoscape (Version 3.9.1).

DAVID, a database for annotation, visualization, and integrated discovery, assists investigators in comprehending the biological significance behind extensive gene lists.^[29]^ This resource was employed to conduct Gene Ontology (GO) enrichment analysis and Kyoto Encyclopedia of Genes and Genomes (KEGG) pathway enrichment analysis for potential targets, using a *P* value < .05 as the cutoff criterion. The resulting data was further visualized through the use of https://www.bioinformatics.com.cn (last accessed on 20 Feb 2023), an online platform dedicated to data analysis and visualization.

### 2.3. Molecular docking and molecular dynamic simulation

#### 2.3.1. Screening ligands from components of the core CMs-pair.

The chemical components of the core CMs-pair were acquired as “2.2. Potential Active Mechanism of the Core CM-Pair.” Upon analyzing the CM-component-target network, 2 core components—Quercetin and Celabenzine—were chosen as ligands for molecular docking. Subsequently, these core components’ 3D sdf format structures were downloaded from PubChem (https://pubchem.ncbi.nlm.nih.gov/).

#### 2.3.2. Preparation of target proteins and the active site.

**Figure 2.**
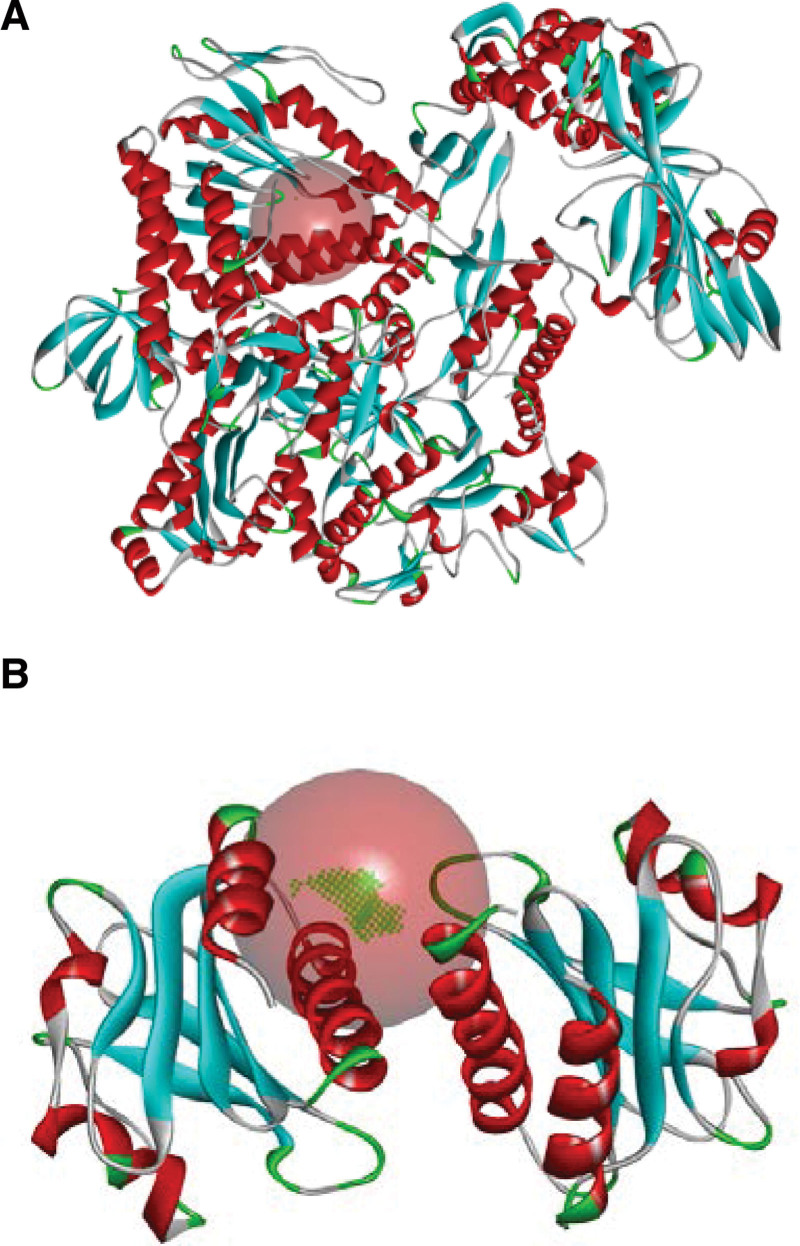
(A) High-resolution crystal structure of MPXV DNA polymerase holoenzyme (PDB-ID: 8HG1). The circle in the figure indicates the active pocket’s position. (B) High-resolution crystal structure of MPXV profilin-like protein A42R (PDB-ID: 4QWO). The circle in the figure indicates the active pocket’s position. MPXV = Mpox virus.

The high-resolution crystal structure of the MPXV DNA polymerase holoenzyme was procured from the PDB (PDB-ID: 8HG1)^[30]^ (Fig. 2A). The holoenzyme structure, in complex with DNA, was determined using cryo-electron microscopy at a global resolution of 2.8 Å. The holoenzyme’s active site was centered on the active amino acid site of the original ligand within the crystal structure. Consequently, the corresponding “active pocket” was constructed. The system searched for the “active pocket” near the active site, ultimately defining 123.518448, 150.473897, and 136.654448 with a radius of 10.62053 Å as the active pocket.

The high-resolution crystal structure of the MPXV profilin-like protein A42R was obtained from the PDB (PDB-ID: 4QWO) (Fig. 2B). The A42R structure was determined using X-ray diffraction at a resolution of 1.52 Å. The protein’s active site was centered on the active amino acid site of the original ligand within the crystal structure. The system searched for the “active pocket” near the active site, and ultimately, 7.924531, −4.299857, and 18.107119 with a radius of 10 Å were defined as the active pocket.

#### 2.3.3. Molecular docking method.

Discovery Studio, a highly visible commercial software integrated by BIOVIA for life science research, encompasses ligand and receptor interaction modules.^[31]^ The Libdock module of Receptor-Ligand Interactions in Discovery Studio 2019 was employed to investigate the binding mechanism between the core components in the core CMs-pair and the key targets of MPXV. Libdock has fast computation speed and the “Libdock Score” can measure the docking results of different molecules and conformations with protein receptors. The higher the “ Libdock Score,” the better the docking. Then, the binding free energy is estimated between each component and the target. The free energy of binding for a receptor-ligand complex can be calculated from the free energies of the complex, the receptor, and the ligand. Using CHARMm-based energies and implicit solvation methods it is possible to estimate these free energies and thus calculate an estimate for the overall binding free energy.

#### 2.3.4. Molecular dynamic simulation method.

The molecular dynamic simulation was carried out using the complex obtained by molecular docking as the initial structure in the Standard Dynamics Cascade module of Discovery Studio 2019. In the simulation process, the molecular parameters of the complex were solvated by using the CHARMM forcefield. A total of 1000 steps of system energy minimization (500 steepest descent method and 500 conjugate gradient method) were completed to achieve the initial equilibrium structure, followed by 50ps of heating (temperature from 50K to 300K), and then 500ps of equilibrium at 300K. The time steps were set to 2fs. When all the thermodynamic parameters are stable, the molecular dynamics simulation is carried out for 10ns with a time step of 2fs and a total number of steps of 5 × 10^7^, saving the results every 10ps and getting a total of 1000 frames. Then, the structural characteristics of molecular dynamics trajectory were analyzed by Analyzing Trajectory module to evaluate the stability of the complex.

This study didn’t require an ethical review, so ethical approval wasn’t necessary.

## 3. Results

### 3.1. Screening results of the selected prescriptions

**Figure 3.**
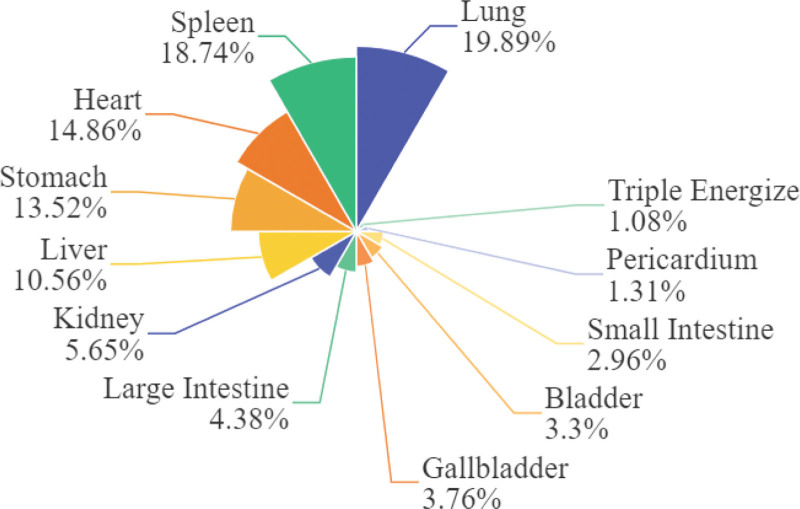
The meridian distribution of CMs. CMs = Chinese Medicines.

Chinese medicine prescriptions (fang ji in Chinese) served as the primary form of clinical practice in TCM. In our study, we selected 119 prescriptions encompassing 109 types of Chinese Medicines (CMs) from Exclusive Methods for Treating Pox (Dou Zhen Xin Fa) for smallpox treatment. Our findings revealed that in these 119 prescriptions, 42.23% of CMs possessed cool/cold properties, 41.18% had warm/heat properties, and 16.59% exhibited neutral properties. Sweet, bitter, or pungent CMs accounted for 90.63% of the total, with sweet CMs being the most frequent, followed by bitter and pungent CMs. Meridian affinity analysis results showed that 19.89% of CMs belonged to the lung meridian, while 18.74%, 14.86%, and 13.52% were associated with the spleen, heart, and stomach meridians, respectively. The results are presented in Table 1 and Figure 3.

**Table 1:** The properties and flavors of CMs in the prescriptions.

Properties	Frequency	Proportion (%)	Flavor	Frequency	Proportion (%)
Cold	340	39.44	Sweet	438	33.08
Warm	340	39.44	Bitter	384	29.00
Neutral	143	16.59	Pungent	378	28.55
Cool	24	2.78	Bland	53	4.00
Heat	15	1.74	Sour	47	3.55
			Salty	19	1.44
			Astringent	5	0.38

CMs = Chinese Medicines.

The Ming Dynasty marked the zenith of TCM development, featuring numerous renowned physicians. Wan Quan, a representative pediatrician of the era, devised these prescriptions, reflecting his approach to smallpox treatment: a combination of tonification and purgation, cold and warm treatments, adaptability based on the disease’s progression, and a focus on achieving therapeutic balance. This methodology could be traced back to the thoughts and experiences of previous generations of doctors. Ge Hong, during the Eastern Jin Dynasty, first documented smallpox in his work, Emergency Prescriptions Reserved Behind the Elbow (Zhou Hou Bei Ji Fang in Chinese). In the Song Dynasty, a relatively comprehensive diagnosis and treatment system for smallpox was established amid the academic contention between the Cold and Warm Schools. Qian Yi, representing the Cold School, excelled in treating smallpox with cold and cool CMs, while Chen Wenzhong, a representative of the Warm School, was adept at using warm and tonic CMs for smallpox treatment. Wan Quan integrated their thoughts and formed his academic system.

### 3.2. High-frequency CMs from the selected prescriptions

In order to identify high-frequency CMs, we employed a frequency analysis method to count the occurrence of CMs in the selected prescriptions. The results revealed 25 types of CMs with the highest frequency in these prescriptions (Table 2). Gancao was the most frequently used, followed by Renshen and Danggui, both of which appeared more than 30 times. Among these high-frequency CMs, ten exhibited warm/heat properties, 12 possessed cool/cold properties, and 3 displayed neutral properties. The frequency distribution of CM properties reflected the treatment philosophy of emphasizing both cold and warm properties equally.

**Table 2:** The high-frequency CMs from prescriptions (top 20).

No.	Chinese name	Plant name*	Part used	Frequency	Proportion (%)
1	Gancao	*Glycyrrhiza uralensis* Fisch.	Rhizome	87	10.22
2	Renshen	*Panax ginseng* C. A. Mey.	Root	40	4.70
3	Danggui	*Angelica sinensis* (Oliv.) Diels	Root	34	4.00
4	Shengjiang	*Zingiber officinale* Roscoe	Rhizome	27	3.17
5	Baishao	*Paeonia* L.	Root	27	3.17
6	Fangfeng	*Saposhnikovia divaricata* (Turcz.) Schischk.	Root	25	2.94
7	Fuling	*Wolfiporia cocos* F.A.Wolf	Sclerotium	23	2.70
8	Jiegeng	*Platycodon grandiflorus* A.DC.	Root	21	2.47
9	Shengma	*Cimicifuga foetida* L.	Rhizome	20	2.35
10	Huangqin	*Scutellaria baicalensis* Georgi	Root	20	2.35
11	Chaihu	*Bupleurum chinense* DC.	Root	19	2.23
12	Chuanxiong	*Ligusticum* L.	Root	18	2.12
13	Huangqi	*Astragalus mongholicus* Bunge	Root	17	2.00
14	Dihuang	*Rehmannia glutinosa* (Gaertn.) DC.	Root	17	2.00
15	Dahuang	*Rheum officinale* Baill.	Rhizome	16	1.88
16	Zhizi	*Gardenia jasminoides* J.Ellis	Fruit	15	1.76
17	Mutong	*Akebia quinata* (Thunb. ex Houtt.) Decne.	Stem	15	1.76
18	Baizhu	*Atractylodes macrocephala* Koidz.	Rhizome	15	1.76
19	Lianqiao	*Forsythia suspensa* Vahl	Fruit	14	1.65
20	Chenpi	*Citrus reticulata* Blanco	Fruit	14	1.65
21	Zicao	*Lithospermum erythrorhizon* Siebold & Zucc.	Root	12	1.41
22	Niubangzi	*Arctium nemorosum* Lej.	Fruit	12	1.41
23	Muxiang	*Aucklandia lappa* Decne.	Root	12	1.41
24	Jingjie	*Schizonepeta tenuifolia* Briq.	Aerial	12	1.41
25	Huanglian	*Coptis chinensis* Franch.	Rhizome	12	1.41

CMs = Chinese Medicines.

* The English names by “ WFO. World Flora Online. Published on the Internet. 2023. Available at: http://www.worldfloraonline.org [access date April 21, 2023].”

Emerging evidence suggested that the host immune response to MPXV played a crucial role in disease pathogenesis and clinical manifestations. The MPXV-mediated immune injury lead to poor clinical outcomes in patients with MPX.^[32]^ CMs with warm/heat properties had the effect of supplementing qi and nourishing blood, which could enhance immunity against infection.^[33,34]^ Examples included Renshen, Danggui, Huangqi, and Baizhu. CMs with cool/cold properties exerted effects of clearing heat and eliminating evil, which could be anti-inflammatory and antiviral. Examples included Shengma, *Scutellaria baicalensis* Georgi (Huangqin), *Rheum officinale* Baill. (Dahuang), and Zicao. These results highlighted the principle of TCM in strengthening the body’s resistance to eliminate pathogenic factors, which could alleviate symptoms and address the root cause of the MPX disease.

### 3.3. High-frequency CM pairs from the selected prescriptions

**Figure 4.**
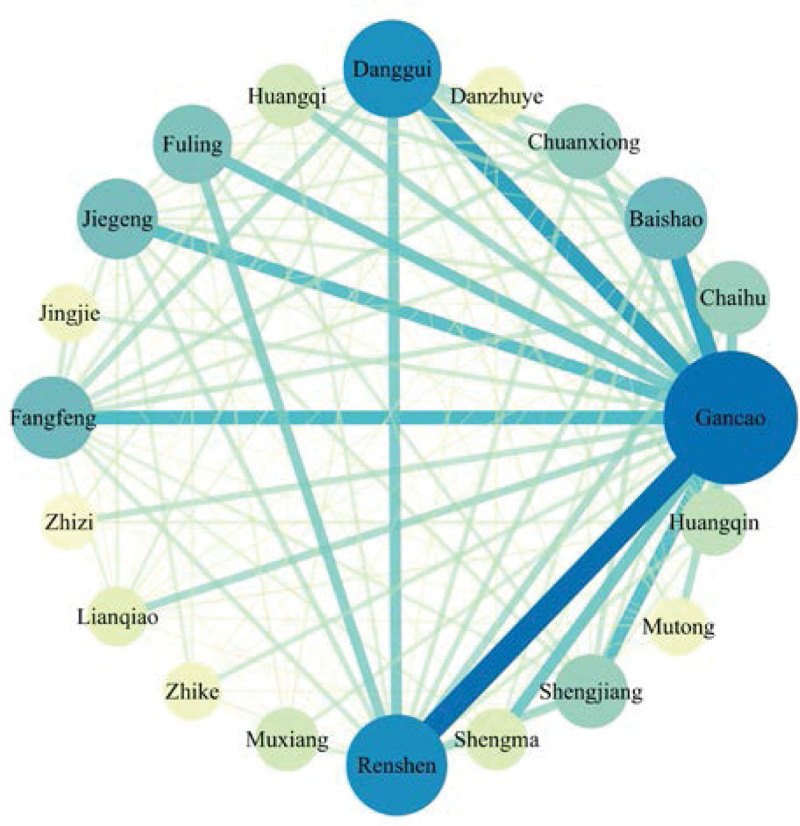
The association network of CMs from selected prescriptions (degree of node ≥ 24). CMs = Chinese Medicines.

CM pairs were a critical aspect of CM compatibility,^[35]^ a unique combination of 2 relatively fixed herbs in the clinic.^[36]^ To identify high-frequency CM pairs in the selected prescriptions, we used SPSS Modeler 18.0 software to construct a CM association network, which was subsequently visualized using Cytoscape 3.9.1. In this network, CMs with degree values greater than twice the median (degree = 24) were depicted in Figure 4. In Figure 4, nodes represented CMs, and lines represented correlations between CMs. The larger and more colorful the node, the more prominent its role in the network. The thicker and darker the lines, the stronger the correlations between CMs. The figure demonstrates that Gancao, Renshen, and Danggui were more significant in the network and had stronger correlation relationships.

Furthermore, the results identified 23 CM pairs with a frequency of more than 10% in the selected prescriptions (Table 3). Gancao was the most commonly used herb in high-frequency CM pairs, followed by Renshen and Danggui. The most frequently used CM pair was the Gancao and Renshen pair, which served as the primary herb in the Si-Jun-Zi decoction. Renshen was a natural resource with shared medicinal and food origins, and it was widely employed in clinical settings for its vital energy-reinforcing effects. Meanwhile, Gancao had the properties of tonifying the middle body and supplementing Qi. The Gancao and Renshen pair could be utilized to treat deficiency syndromes caused by MPXV.

**Table 3:** High-frequency CM-pair in prescriptions (proportion > 10%).

No.	CM-pair	Frequency	Proportion (%)
1	Gancao&Renshen	35	29.41
2	Danggui&Gancao	25	21.01
3	Baishao&Gancao	25	21.01
4	Fangfeng&Gancao	21	17.65
5	Gancao&Jiegeng	20	16.81
6	Gancao&Shengjiang	20	16.81
7	Gancao&Shengma	18	15.13
8	Fuling&Gancao	18	15.13
9	Danggui&Renshen	17	14.29
10	Chaihu&Gancao	16	13.45
11	Fuling&Renshen	16	13.45
12	Renshen&Shengjiang	14	11.76
13	Gancao&Huangqin	14	11.76
14	Gancao&Huangqi	14	11.76
15	Chaihu&Renshen	13	10.92
16	Chuanxiong&Gancao	13	10.92
17	Chuanxiong&Danggui	13	10.92
18	Gancao&Lianqiao	13	10.92
19	Gancao&Mutong	13	10.92
20	Dihuang&Gancao	13	10.92
21	Baishao&Danggui	12	10.08
22	Baizhu&Gancao	12	10.08
23	Danggui&Fangfeng	12	10.08

CMs = Chinese Medicines.

### 3.4. Analysis of association rules of CMs from the selected prescriptions

Association rules refer to specific relationships between the values of two or more variables. These rules do not imply causality but often exhibit phenomena of “coexistence or “inferring one object from another.”^[37]^ As a major data mining method, association rules are increasingly applied in the field of TCM to study the compatibility of CM prescriptions. Using SPSS Modeler 18.0 software with the Apriori algorithm, the association rule of CMs from the selected prescriptions was analyzed based on the criteria of “support ≥ 10.0%, confidence ≥ 80.0%, and a maximum of 2 antecedents.”

The results of the association rule analysis, presented in Tables 4 and 5, revealed that Gancao, Renshen, and Danggui are essential CMs in the selected prescriptions. Danggui possessed properties that nourish blood and activate blood circulation, which could enhance compatibility when combined with Gancao and Renshen.

**Table 4:** Results of 2-term association rule analysis of herbs.

No.	Consequent	Antecedent	Support degree (%)	Confidence degree (%)
1	Gancao	Renshen	33.61	87.50
2	Gancao	Baishao	22.69	92.59
3	Gancao	Fangfeng	21.01	84.00
4	Gancao	Jiegeng	17.65	95.24
5	Gancao	Shengma	16.81	90.00
6	Gancao	Chaihu	15.97	84.21
7	Gancao	Huangqi	14.29	82.35
8	Gancao	Mutong	12.61	86.67
9	Gancao	Baizhu	12.61	80.00
10	Gancao	Lianqiao	11.77	92.86

**Table 5:** Results of 3-term association rule analysis of herbs.

No.	Consequent	Antecedent	Support degree (%)	Confidence degree (%)
1	Gancao	Danggui, Renshen	14.286	82.353
2	Gancao	Fuling, Renshen	13.445	81.25
3	Gancao	Chaihu, Renshen	10.924	84.615
4	Renshen	Baizhu, Gancao	10.084	83.333
5	Gancao	Baishao, Danggui	10.084	91.667

### 3.5. Systemic pharmacological analysis of the core CM-pair

**Figure 5.**
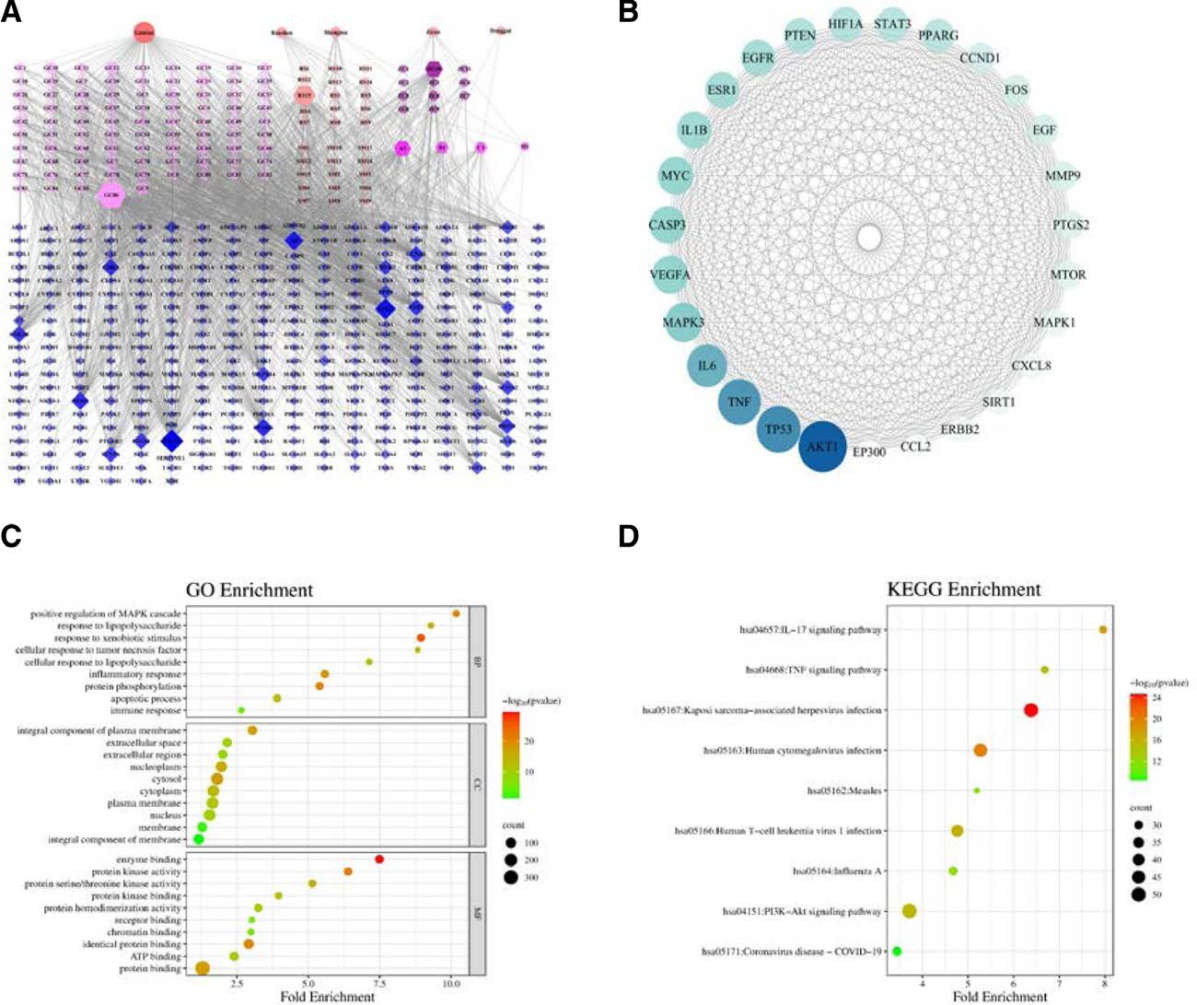
(A) The herb-component-target network of the 5 core herbs. Nodes representing herbs are depicted as circles, components as hexagons, and targets as diamonds. (B) PPI network of key CM-pair targets (degree > 100). (C) GO enrichment analysis results for candidate targets. (D) KEGG pathway enrichment analysis results for candidate targets. CMs = Chinese Medicines, GO = Gene Ontology, KEGG = Kyoto Encyclopedia of Genes and Genomes, PPI = protein–protein interaction.

The results of the frequency analysis showed that Gancao, Renshen, and Danggui were the 3 most frequently used CMs. The results of the association network and association rules showed that Gancao, Renshen, and Danggui were CMs with strong correlations. What’s more, Gancao, Renshen, and Danggui were the representative CMs with warm/heat properties, which were common CMs for treating smallpox. Traditional Chinese medicine emphasizes the combination of cold and warm in the treatment of smallpox. So, some representative cool/cold CMs were equally important. Among the cool/cold CMs with a high frequency of use, Shengma and Zicao had an excellent effect on promoting eruption. They were very commonly used CMs for the treatment of smallpox in clinics which were worthy of further investigation. Based on the data mining results and clinical experience, Gancao, Renshen, Danggui, Shengma, and Zicao were selected as the core herbs for treating smallpox. These herbs might also hold referential value for treating MPX. Gancao, Renshen, and Danggui were representative herbs with warm/heat properties, while Shengma and Zicao exhibited cool/cold properties and promoted eruption, which could be beneficial for treating excess syndrome caused by MPXV. In this study, we identified 131 active components from the 5 core herbs and 348 potential targets for these components. A herb-component-target network was constructed to visually display the intricate relationships among herbs, components, and targets (Fig. 5A). Among these active components, those with high interconnection degrees played more significant roles in the network. Following the criteria of “degree ≥ 30,” we screened 8 components, as shown in Table 6. Additionally, we constructed a PPI network for the component targets, comprising 341 nodes and 6568 edges. According to “degree ≥ 100,” we identified 27 key targets (Fig. 5B). The 348 targets were imported into DAVID for GO and KEGG pathway enrichment analysis. The GO enrichment analysis yielded 243 molecular function items, 1087 biological process items, and 141 cellular component items, while the KEGG pathway enrichment analysis identified a total of 191 pathways. The significant results related to MPX are depicted in Figure 5C and D. The findings suggest that the core CM-pair exerts effects on the immune system, endocrine system, inflammatory response, and viral infections, such as measles, COVID-19, and influenza A. Consequently, we hypothesize that the Gancao, Renshen, Danggui, Shengma, and Zicao CM-pair produce therapeutic effects in the treatment of MPX by targeting multiple pathways and molecular targets in the human body through their complex active components.

**Table 6:** The core components of the core CMs-pair (degree ≥ 30).

Herb	Abbreviation	Component	PubChem CID	Degree
Gancao	GC87	Quercetin	5280343	145
Renshen	RS15	Celabenzine	442847	95
Zicao	ZC10	Des-O-methyllasiodiplodin	14562695	66
Gancao, Renshen	A1	Kaempferol	5280863	59
Gancao	GC12	Karingenin	439246	35
Renshen, Danggui	B1	Beta-itosterol	222284	33
Gancao	GC10	Formononetin	5280378	32
Gancao	GC7	Isorhamnetin	5281654	30

CMs = Chinese Medicines.

### 3.6. Molecular docking patterns of the core components in the core CM-pair with MPXV targets

**Figure 6.**
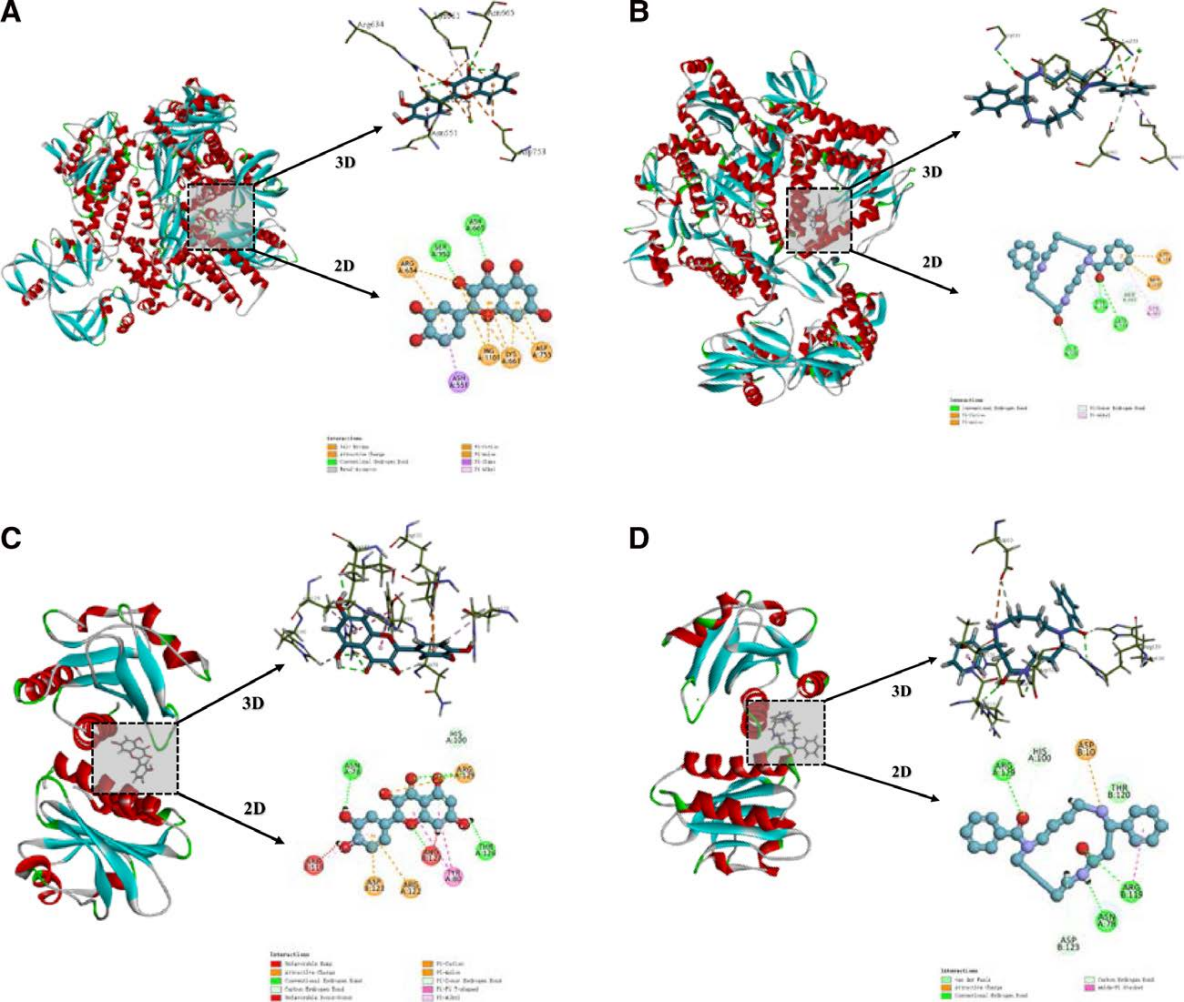
Molecular docking patterns of core components with MPXV target. (A) Quercetin with 8HG1; (B) Celabenzine with 8HG1; (C) Quercetin with 4QWO; and (D) Celabenzine with 4QWO. MPXV = Mpox virus.

According to the results of “3.5 Systemic Pharmacological Analysis of the Core CM-Pair,” two core components, quercetin, and celabenzine, were selected as ligands for molecular docking with the high-resolution crystal structure of the MPXV DNA polymerase holoenzyme (PDB-ID: 8HG1) and the profilin-like protein A42R(PDB-ID:4QWO). The molecular docking results showed good docking activity and the molecular docking poses with the highest Libdock score are illustrated in Figure 6. The binding energy reflected the possibility of binding between the receptor and ligand. The lower the binding energy, the higher the affinity between the receptor and ligand, and the more stable the conformation, indicating that the lower the released energy of the 2 molecules in the natural state, the easier it was to bind. It was generally agreed that binding energy<- 4.25 kcal/mol indicates binding activity, binding energy < -5 kcal/mol indicates good binding activity and binding energy < -7 kcal/mol indicates that it has strong binding activity.^[38]^ The result showed that all of the molecular docking patterns had bind energy＜7kcal/mol, indicating strong binding activity.

The DNA polymerase holoenzyme had been confirmed to play a crucial role in the DNA replication processivity of MPXV, and it might guide the development of anti-poxvirus drugs. Among the 2 selected compounds, celabenzine exhibits a higher Libdock score with the target. In celabenzine, conventional hydrogen bonds were formed with GLY531, TYR554, and TEU553, and mixed Pi/Alkyl hydrophobic interactions were formed with LYS661 and TYR554. In quercetin, conventional hydrogen bonds were formed with SER552 and ASN665, and mixed Pi/Alkyl hydrophobic interactions were formed with ASN551 and LYS661.

The profilin-like protein A42R was a short protein encoded by the MPXV virus gp153 locus, displaying sequence similarity to eukaryotic profilins, which were actin-binding proteins involved in the regulation of cytoskeletal structure and function.^[39,40]^ Studies had shown that A42R may engage in distinct interactions with phosphatidylinositol lipids. Although the role of A42R in MPXV virus replication was not yet fully understood, it could also be selected as a therapeutic target for structure-based drug design. Among the 2 selected components, quercetin exhibited a higher Libdock score with A42R. In quercetin, conventional hydrogen bonds were formed with ASN78, THR126, ARG127, and ARG129, and mixed Pi/Alkyl hydrophobic interactions were formed with ARG129 and ARG119. In celabenzine, conventional hydrogen bonds were formed with ARG129, ARG119, and ASN78. These interaction sites were important factors to consider in studying molecular structure-activity relationships and designing new drugs at the molecular level.

**Figure 7.**
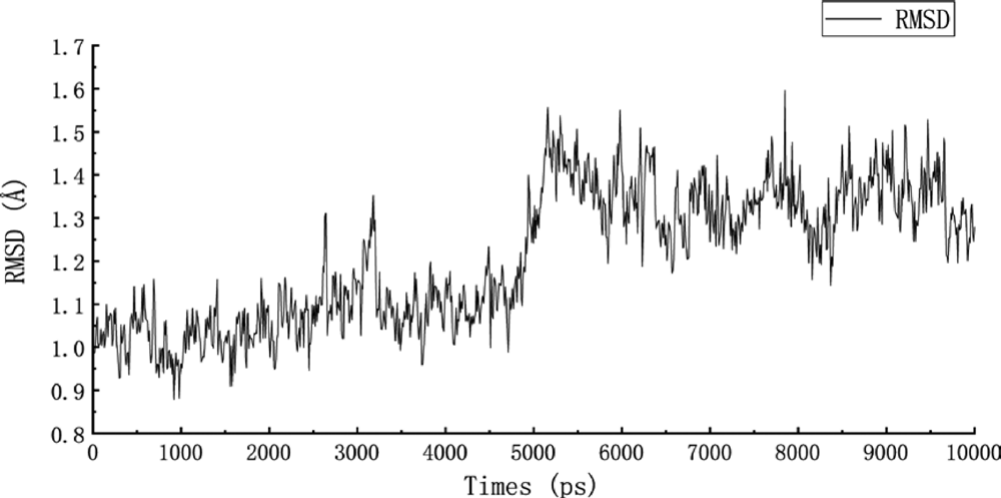
RMSD changes during the binding of quercetin with 4QWO.

**Figure 8.**
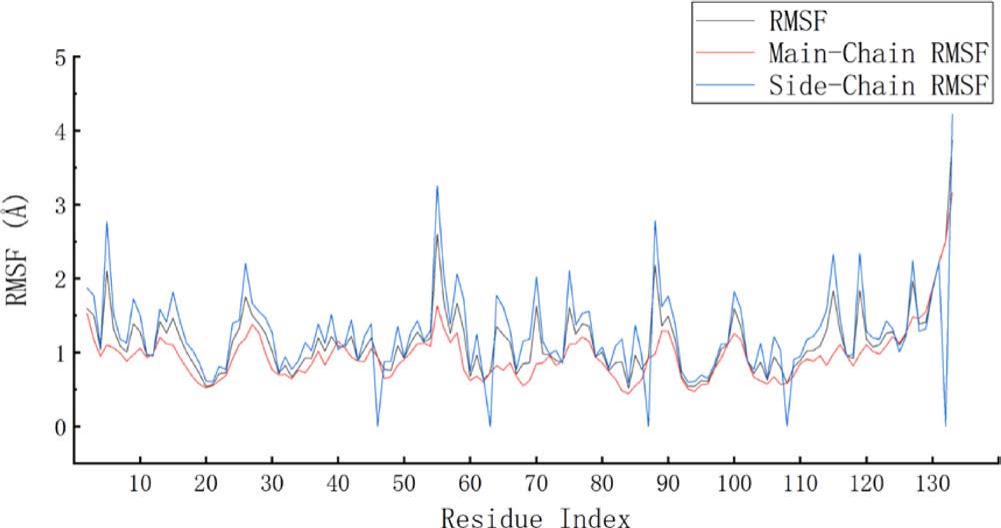
RMSF changes during the binding of quercetin with 4QWO.

Molecular dynamic simulation was a computer experimental method to calculate the macroscopic properties of molecules by simulating the motion of microscopic particles on the basis of a molecular model.^[41,42]^ Molecular dynamic simulation could be used to study the stability of complexes. In this study, we selected quercetin and 4QWO, with better binding properties, for further molecular dynamic simulation. We calculated RMSD and RMSF in the simulation process to evaluate the stability of the complex. RMSD could reveal the position changes between the protein conformation and the initial conformation in the simulation process. RMSD fluctuated from frame to frame with an average value (0–0.8 Å), indicating that the system had reached equilibrium, and although there were structural differences between conformations, the differences were small and the trajectory was stable.^[43]^ RMSF showed the fluctuation of amino acid residues relative to the average structure of the trajectory, which could characterize the flexibility and motion intensity of amino acids in the whole simulation process. The smaller RMSF indicated the more convergent and stable the system.^[44]^ The RMSD and RMSF changes during the binding of quercetin with 4QWO were shown in Figures 7 and 8. It could be seen that the RMSD of the complex has an obvious fluctuation at about 5ns, and then showed a stable motion state in the rest of the simulation process, consistently maintaining fluctuations within 1.6 Å, with a fluctuation range of less than 0.8 Å. This indicated that the dynamic binding of quercetin and 4QWO was in a relatively stable state. The RMSF changes showed that the RMSF of the main chain is smaller than the side chain. Although the RMSF of the residues at both ends of the protein was large, it is far from the residue at the ligand binding active site. On the contrary, the RMSF of the residues at the active site (such as residues ARG119, ASP123, LEU106, ILE96, and GLU83) fluctuated little, indicating that the binding of the protein to the ligand promoted the stability of the spatial structure of the protein, which further indicated the stability of the binding of the ligand to the protein.

## 4. Discussion and Conclusions

The clinical manifestations of monkeypox are similar to smallpox. The most prevalent clinical manifestations include rash, fever, pruritus, pruritus, and lymphadenopathy. Other manifestations include fatigue, sore throat, headache, cough, myalgias, photophobia, arthralgia, difficult breathing, conjunctivitis, nausea/vomiting, and diarrhea.^[45]^ For thousands of years, under the guidance of the “holistic concept and syndrome differentiation” in TCM, Chinese medicines and their prescriptions have demonstrated multi-effect synergistic effects on infectious diseases such as smallpox. In modern society, their indispensable roles have been confirmed in SARS, H1N1 influenza epidemics, and COVID-19.^[46–48]^ In the current MPX epidemic, Sheng-Ma-Ge-Gen decoction, Sheng-Jiang-San, and Zi-Xue-San were recommended for patients with fever, while Qing-Ying decoction, Sheng-Ma-Bie-Jia decoction, and Xuan-Bai-Cheng-Qi decoction were recommended for patients with high fever, acne, sore throat, and multiple swollen and painful lymph nodes by the National Health Commission and the National Administration of TCM of the People’s Republic of China. These are all ancient, classic, and famous TCM prescriptions. Thus, it is crucial to have a more in-depth analysis of these prescriptions and their mechanisms of action.

To analyze the rules of Chinese medicines in treating smallpox and gain insights into the treatment of MPX, we selected 119 prescriptions from a medical book written by a renowned Ming dynasty doctor. We identified 25 high-frequency Chinese medicines and 23 high-frequency Chinese medicine pairs among these prescriptions. Combined with the results of the association rule analysis, Gancao, Renshen, Danggui, Shengma, and Zicao were selected as the core Chinese medicine pair for further study. Gancao is one of the most widely used CMs in the world, with the following actions: supplementing spleen qi, clearing heat and relieving toxicity, dispelling phlegm and relieving cough, relaxing tension and relieving pain, and harmonizing the nature of other herbal medicines,^[49]^ indicated for fatigue, cough and phlegm, myalgia, and difficult breathing caused by MPXV. Renshen was the most revered of the herbs in ancient times in China, Korea, Japan, and America,^[50]^ with the following actions: reinforcing vital energy, tonifying visceral qi, calming mind and benefiting intelligence, and helping produce saliva and slaking thirst, indicated for qi deficiency syndrome caused by MPXV. Danggui was usually used to invigorate blood circulation in the treatment of menstrual disorders,^[51]^ with the following actions: tonifying blood, promoting blood circulation, regulating menstruation, relieving pain, and moistening intestines, indicated for blood stasis and blood deficiency syndrome caused by MPXV. The combination of the Gancao, Renshen, and Danggui can play a synergistic effect of tonifying qi and blood and expressing toxin outward, which is suitable for deficiency syndrome caused by MPXV without the typical clinical manifestations and difficult to cure. What’s more, modern pharmacological studies had shown that they had the functions of antiviral, anti-inflammatory, anti-fatigue, and promoting immune-modulating,^[52–54]^ which had certain effects in treating deficiency syndrome of MPX. Shengma was the main herb of Sheng-Ma-Ge-Gen Docotion, with the following actions: evacuating wind-heat, lifting yang qi, clearing heat and detoxifying, and promoting eruption, indicated for wind-heat syndrome or heat-toxin syndrome caused by MPXV. Zicao was a key herb for treating smallpox, with the following actions: clearing heat and cooling blood, promoting blood circulation, detoxifying, and promoting eruption, indicated for blood-heat syndrome or heat-toxin syndrome caused by MPXV. The Shengma and Zicao pair is suitable for excess heat syndrome caused by MPXV with typical clinical manifestations. And modern pharmacological studies had also shown that they had the functions of antiviral, anti-inflammatory, immune regulation, and easing pain,^[55,56]^ which had certain effects in treating the excess heat syndrome of MPX. However, CMs had strict discipline on dosage and duration, otherwise, they will produce side effects. Studies had found that Gancao had salt corticosteroid components. Excessive Gancao consumption could lead to hyper mineralocorticoidism, a condition marked by salt retention, hypokalemia, hypertension, metabolic alkalosis, hypoaldosteronism, and low renin activity, leading to significant life-threatening consequences, particularly in individuals with cardiovascular disease.^[57,58]^ Due to its action of decreasing blood glucose, Renshen should be used with caution for patients with low coagulation function.^[59]^ The most common side effects of Renshen resulting from its overdose were nervousness and excitability-- the inability to sleep and high blood pressure.^[60]^ Although Danggui was usually well tolerated in general use, it might increase the risk of bleeding in patients taking warfarin and antiplatelet drugs and cause photosensitivity reactions.^[61]^ Shengma and Zicao are relatively safe CMs, but long-term use will lead to liver and kidney damage. Due to their bitter taste and cold property, they easily affected digestive function, so they are generally used in the acute phase and are rarely used after symptom relief.

In the systematic network pharmacology analysis, we obtained 131 active components and 348 candidate targets of the core Chinese medicine pairs. We then constructed the herb-component-target and PPI networks. By analyzing the network topology, 2 core components, quercetin and celabenzine, were selected for molecular docking analysis. Quercetin was isolated from the hydroalcoholic extract of *Humulus lupulus* L. This extract was found to inhibit replication of various viral strains, at a different time from infection.^[62]^ And, quercetin was reported to inhibit viral neuraminidase activities in vitro and influenza infection in animal models.^[63]^ Celabenzine was a natural product found in Gymnosporia mossambicensis and Tripterygium wilfordii. Tripterygium wilfordii had significant anti-inflammatory and immune-modulating and is widely used in various autoimmune-mediated inflammatory diseases, including rheumatoid arthritis, nephrotic syndrome, systemic lupus erythematosus, and Behcet’s disease.^[64]^ The GO and KEGG pathway enrichment analysis revealed that the core Chinese medicine pairs exerted effects on the immune system, endocrine system, inflammatory response, and viral infections. MPX induces a robust immune response, internal environment disorder, and an inflammatory storm, during which numerous cytokines are activated. These results showed that the core Chinese medicine pair may regulate immune-related and inflammation-related pathways to alleviate and eliminate symptoms. Furthermore, the molecular docking and molecular dynamics simulation results demonstrated the binding mechanisms between the selected components and the known MPXV targets, indicating potential anti-MPXV virus activity.

In conclusion, by combining the knowledge of ancient prescriptions with modern pharmacological research methods, we analyzed the compatibility of Chinese medicines for treating smallpox and explored the potential molecular mechanisms of the key herbs for addressing MPX. These findings underscored the characteristic multi-component and multi-target nature of Chinese medicines. However, it is important to note that virtual screening results have inherent limitations. Therefore, further in vitro and in vivo experiments are needed to validate the findings of this study in later stages. This research aims to provide a solid experimental foundation for the development of natural antiviral drugs.

## Author contributions

**Conceptualization:** Haozhong Wang.

**Data curation:** Chengchen Xu.

**Formal analysis:** Linyang LI.

**Funding acquisition:** Yinling Guo.

**Investigation:** Linyang LI.

**Methodology:** Linyang LI, Haozhong Wang.

**Project administration:** Yinling Guo, Haozhong Wang.

**Resources:** Linyang LI.

**Software:** Linyang LI, Chengchen Xu.

**Supervision:** Linyang LI, Haozhong Wang.

**Validation:** Linyang LI, Chengchen Xu.

**Visualization:** Linyang LI.

**Writing – original draft:** Linyang LI.

**Writing – review & editing:** Linyang LI, Chengchen Xu, Haozhong Wang.
